# Decentralized dynamic understanding of hidden relations in complex networks

**DOI:** 10.1038/s41598-018-19356-4

**Published:** 2018-01-25

**Authors:** Decebal Constantin Mocanu, Georgios Exarchakos, Antonio Liotta

**Affiliations:** 1 Department of Mathematics and Computer Science, Eindhoven University of Technology, Eindhoven, 5612 AP The Netherlands; 2 Department of Electrical Engineering, Eindhoven University of Technology, Eindhoven, 5612 AP The Netherlands; 3 Present Address: Department of Electronics, Computing and Mathematics, University of Derby, Derby, DE22 1GB UK

## Abstract

Almost all the natural or human made systems can be understood and controlled using complex networks. This is a difficult problem due to the very large number of elements in such networks, on the order of billions and higher, which makes it impossible to use conventional network analysis methods. Herein, we employ artificial intelligence (specifically swarm computing), to compute centrality metrics in a completely decentralized fashion. More exactly, we show that by overlaying a homogeneous artificial system (inspired by swarm intelligence) over a complex network (which is a heterogeneous system), and playing a game in the fused system, the changes in the homogeneous system will reflect perfectly the complex network properties. Our method, dubbed Game of Thieves (GOT), computes the importance of all network elements (both nodes and edges) in polylogarithmic time with respect to the total number of nodes. Contrary, the state-of-the-art methods need at least a quadratic time. Moreover, the excellent capabilities of our proposed approach, it terms of speed, accuracy, and functionality, open the path for better ways of understanding and controlling complex networks.

## Introduction

In any real-world system, at micro and macro-scale, from the vigintillions of interacting atoms in the observable universe, to the billions of persons who live on Earth, there are amazing structures of networks of networks. These networks can be studied, understood, and controlled by the means of network science and complex networks^1^, leading to advances in many domains, including neuroscience^2–4^, astrophysics^5^, biology^6,7^ epidemiology^8^, social networks^9,10^, transportation networks^11^, communication networks^12,13^, and artificial intelligence^14^ (to mention but a few). Yet, unveiling the complex networks hidden patterns and computing even their most basic properties is far from trivial, due to the massive number of node entangles that interact in non-obvious ways, evolving and unfolding continuously^15^.

Among all these network properties, the centrality (or importance) of nodes and links is fundamental to understanding things such as: biological neural networks^2–4^, cosmic structures^5^, biological networks^7^, how viruses spread or can be contained^16^; which people or news are influencing opinions and decisions the most^17^; how to protect computer systems from cyber-attacks^18^; or how to relay data packets in the one-trillion Internet-of-Things network of the future. While there is ample literature on node centrality computation^19^, the existing methods do not scale to the size and dynamics of practical complex networks, which operate at the tunes of millions to trillions nodes. Besides that, the state-of-the-art centrality metrics are designed for specific goals, and one metric which performs well for one goal is suboptimal for another^20^. Furthermore, existing methods focus on finding the most important network elements (i.e. nodes or links), but fail to capture the hidden relations across the whole network links and nodes. The centralized algorithms consider the topology as a whole, overlooking many of the local features^19^.

*Per contra*, the decentralized methods are usually based on local computations to construct statistics of network elements (as in^21^), but fail to capture the overall network structure. In fact, the most effective decentralized methods nowadays still fail to capture all the relations between the networks elements, and this is our main target. In addition, current methods have technological constraints that have to be surpassed. To tackle the scale as well as dynamics of real-world networks, we need to compute centrality metrics not only accurately but also timely, based on the existing computational capabilities.

To tackle all of the above constraints and limitations, in this paper we propose a new viewpoint to model and understand complex networks. The basic idea is fairly simple. First, we overlay a homogeneous artificial system (a system created in such a way that all its elements ponder equally) over a complex network, which is a heterogeneous system - its level of heterogeneity being given by its topology. We then start a gaming process, whereby the artificial system entities start interacting with the network. What’s interesting is the artificial system evolves in different ways, depending on the features of the complex network. In turn, network features, specifically the centrality metrics, start emerging. Our viewpoint is inspired to a basic principle of physics. If one would like to measure the volume of an irregular-shape object then one solution would be analytical, by measuring its dimensions and by solving some complicated triple integrals. An alternative much faster and ingenious solution, which needs just middle school knowledge, is the water displacement method coming from the Ancient Greeks, i.e. Archimedes of Syracuse. One would need just to submerge that irregular object in a graduated cylinder filled with water and to measure the water displacement. Further on, this easy to obtain volume can be used to measure other properties of the object, e.g. density.

Keeping the proportion, in the case of complex networks, the artificial homogeneous system represents the water, and the centrality represents the volume, while the game represents the action of submerging the irregular object. With the complex networks constraints in mind, our proposed homogeneous system follows four stratagems:

1. completely decentralized computations, so that all nodes contribute simultaneously to the calculation of centrality;
2. computational simplicity, so that the algorithm may be executed in thin nodes, such as the low-resources sensors of the Internet of Things;
3. nature-inspired, swarm computations^22^, to pursue global convergence through localized, stochastic actions;
4. human-behaviour like computations^23^(namely, egoistic behaviour), to gain an insight on the topological features of the network.

Altogether, the above four stratagems are confined in a novel algorithm, dubbed Game of Thieves (GOT).

## Results

### Game of Thieves

Intuitively, GOT mimics the egoistic behaviour of a multitude of thieves faced with the prospect of easy-to-steal diamonds - from here comes its name. Our homogeneous artificial system has two virtual elements: a group of wandering thieves (in game theory: the actors) and a set of virtual diamonds or vdiamonds (in game theory: the resources). At start, each node is artificially endowed with vdiamonds which are nomadic, reusable and non-replicable virtual resources, generalizing and virtualizing the concept from^12,24^. Likewise, each node is endowed with wandering thieves, mobile actors which act stochastic (they wander in search of vdiamonds to steal) and egoistic (as soon as they have an opportunity, they steal vdiamonds and take them back to their home node).

A thief has two states: “empty” (i.e. it does not carry any vdiamond) and “loaded” (i.e. it carries one vdiamond). Besides that, he has three functionalities: he wanders from one node to a neighbour, picked randomly (chaotic behaviour), to search for vdiamonds; when he finds vdiamonds, the thief fetches one (egoistic behaviour); he brings it to his home node by following back the same path previously used to find the vdiamond. Like any other vdiamond, this newly homed vdiamond becomes immediately available for the other wandering thieves to steal it. More details about the thieves behavior can be found in Methods. When GOT starts, all nodes host the same given number of thieves and vdiamonds. Then the game proceeds in epochs. At each epoch, all thieves jump from their current location to the next one, changing state when they find or deposit a new vdiamond.

Comparing with classical swarm computational methods, in GOT the thieves do not communicate directly among them - they are independent actors in the game. Nodes, links and thieves perform just local actions, while the interactions at global level are ensured by the vdiamonds migration. In turn, the vdiamonds migration is driven by the network topology (a heterogeneous system), since the resources tend to be drawn more rapidly from the better connected nodes and tend to be accumulated in the less connected nodes. It is through this migration process that the network elements strengths (node and link centralities) gradually emerge from the vdiamonds distribution.

### GOT formalism

Let us consider *G* = (*V*, *E*) to be an undirected graph (*G*) containing a set of nodes (*V*) and a set of edges (*E*). ${{\rm{\Phi }}}_{0}^{n}$ is the initial amount of vdiamonds in node *n* ∈ *V* (at time zero). Similarly, ${{\rm{\Phi }}}_{T}^{n}$ denotes the number of vdiamonds in node *n* ∈ *V* at time *T* (i.e. after the game has run for *T* epochs). ${{\rm{\Psi }}}_{T}^{l}$ is the number of “loaded” thieves traversing link *l* ∈ *E* at epoch *T*. The average number of vdiamonds present at a node (*n*), after the game has run for a duration of *T* epochs, can be computed as:

1 $${\bar{{\rm{\Phi }}}}_{T}^{n}=\frac{1}{T}\sum _{e=0}^{T}{{\rm{\Phi }}}_{e}^{n}$$

The average number of “loaded” thieves passing through link (*l*) after *T* epochs will be:

2 $${\bar{{\rm{\Psi }}}}_{T}^{l}=\frac{1}{T}\sum _{e=0}^{T}{{\rm{\Psi }}}_{e}^{l}$$

Counterintuitively, a smaller ${\bar{{\rm{\Phi }}}}_{T}^{n}$ value reflects a more important node, while a higher ${\bar{{\rm{\Phi }}}}_{T}^{n}$ value indicates a less important one. This is a consequence of the fact that the more central nodes are visited by many thieves which will contribute to their fast depletion, while the less central nodes are visited by few thieves which will not be able to deplete them. Intuitively, higher ${\bar{{\rm{\Psi }}}}_{T}^{l}$ values reflect more important links, while lower ${\bar{{\rm{\Psi }}}}_{T}^{l}$ values point to the less important links.

### GOT functionality illustration

**Figure 1:**
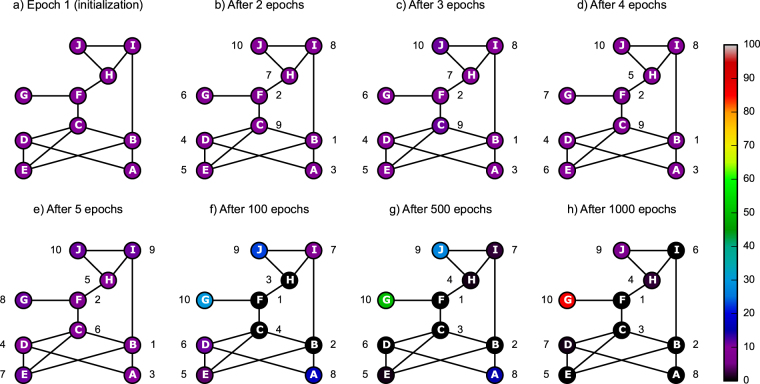
Thieves in action. Snapshots with the illustration of GOT behavior over epochs on a simple unweighted network with 10 nodes. Initially, we set ${{\rm{\Phi }}}_{0}^{n}\mathrm{=10}$ vdiamonds and one thief per node, and we let the game to run for 1000 epochs. The colormap gives the number of vdiamonds, ${{\rm{\Phi }}}_{e}^{n}$, in any node (n) at epoch (e). The numbers on the side of each node show the rank of importance, Λ_*e*,*n*_, assigned by GOT to the specific node (n) after (**e**) epochs, where *e* = 1, 2, 3, 4, 5, 100, 500, and 1000 epochs in subplots **a**,**b**,**c**,**d**,**e**,**f**,**g**, and **h** respectively.

GOT algorithm is presented in Methods, while Fig. 1 shows snapshots of GOT in operation at eight different times, on a simplistic 10-node network. Notably, after just 5 epochs GOT already reflects in a decent manner the nodes centrality. Being a purely stochastic process, GOT rapidly leads to well-organized patterns in the resource distribution, as visible from the evolution of the colour codes over the eight epochs. This behaviour agrees with diffusion-limited aggregation processes^25^ and ensures that the most central nodes lose their resources first (e.g. Figure 1, nodes B, C, F), while the marginal nodes (e.g. Figure 1, node G) will tend to accumulate resources more rapidly (Fig. 1g and h). This also follows the intuition that nodes with higher centrality have higher chances of being visited by thieves. This observation is also compatible with a similar phenomenon discovered by Saavedra *et al*. in the context of real-world biological and economical networks, whereby the strongest network contributors were found to be the most prone to extinction^26^.

### GOT visualization

**Figure 2:**
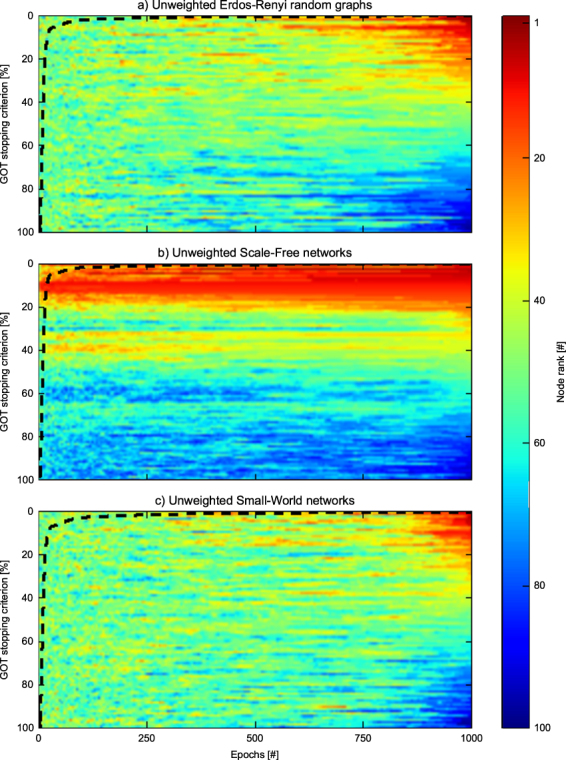
GOT visualization. Nodes rank assigned by GOT in each epoch, while it runs for 1000 epochs in random generated networks with 100 nodes and between 500 and 1000 links. The results are averaged on 10 different networks for each network type. The dash lines show GOT stopping criteria as a percentage of the total number of nodes, at any epoch T.

To begin with, we have tested GOT in small scale simulations, mainly to visualise its operation. We simulated ten Erdös-Rényi Random Graphs^27^, ten Scale-Free networks^28^, and ten Small-World networks^29^, each being unweighted, and including 100 nodes and 500 to 1,000 links. The game started with 1 thief and 100 vdiamonds per node and run for 1,000 epochs. At that point we averaged the results on each network type. Figure 2 shows both the node ranking (following a colour scheme) and GOT’s convergence level (dotted line). Remarkably, after just a few hundred epochs GOT stabilizes, indicating that the striking majority of node ranks have been established. It is interesting to see that scale-free networks stabilize significantly faster (in just a few epochs), as it was expectable by the peculiar node degree distribution on such network types.

### GOT stopping criterion

The stopping criterion of the GOT algorithm is reached when just a small number of nodes still changes their rank of importance from one epoch to the next successive ones using the scores assigned by GOT. Formally, let us define a vector Λ_*e*_ for any epoch (*e*). Each element Λ_*e*,*n*_ ∈ Λ_*e*_ is the rank of importance given by GOT to node *n* ∈ *V* in epoch (*e*). Note that all elements of Λ_*e*_ are unique natural numbers between 1 and |*V*|. Thus, a general stopping criterion for GOT can be expressed as:

3 $$\frac{1}{H}(\sum _{e=T-H}^{T}\sqrt{\sum _{n=1}^{|V|}{({{\rm{\Lambda }}}_{e,n}-{{\rm{\Lambda }}}_{e-1,n})}^{2}}) < \varepsilon |V|$$

where *T* is the actual epoch, *H* is the number of past epochs taken into consideration, and *ε* ∈ (0, 1) is a subunitary real number. Figure 2 reflects GOT stopping criterion for networks with 100 nodes and *H* = 10 over 1000 epochs.

Please note that the GOT stopping criterion from Equation 3 is independent of the order in which the nodes are considered, as long as the same order is used in all epochs. Still, the stopping criterion is dependent on the rank of importance Λ_*e*, *n*_ ∈ Λ_*e*_ assigned by GOT to each node (*n*) in epoch (*e*). As long as the network scrutinized has a heterogeneous topology the GOT algorithm will converge to a statistical equilibrium state. We demonstrate this empirically in the next two paragraphs (i.e. *GOT scalability* and *GOT performance*). Yet, if the network is perfectly homogeneous (e.g. a complete unweighted graph) then GOT will never converge. This behavior, even if it looks unwanted, is in fact as it should be, as in a complete unweighted graph all nodes have exactly the same importance.

In practice, we found that satisfactory results are achieved by setting *H* = 10, and *ε* = 0.02, and we named *SC*_2_ this particular instantiation of Equation 3. In other words, *SC*_2_ means that a maximum 2% of the nodes change their rank over 10 consecutive epochs. We validate the performance of GOT stopped when *SC*_2_ is fulfilled throughout the paper.

### GOT scalability

To study the ability of GOT to scale, we have conducted extensive simulations on a variety of networks, up to one million nodes. We consider three types of randomly generated networks, Erdös-Rényi Random Graphs, Scale-Free and Small-World networks, both weighted and unweighted. Simulations are randomized, repeated and averaged to ensure statistical significance. We look at the number of epochs required for GOT to converge, using the stopping criterion described above (*SC*_2_). Therein we shall also discuss why *SC*_2_ is satisfactory for the assessment of node and link centrality. We simulate networks ranging from 10 to 10^6^ nodes, having a number of links comprised between six and ten times the number of nodes. We also tried different starting conditions, with 1, 3 and 10 thieves per node, setting ${{\rm{\Phi }}}_{0}^{n}=|V|$.

**Figure 3:**
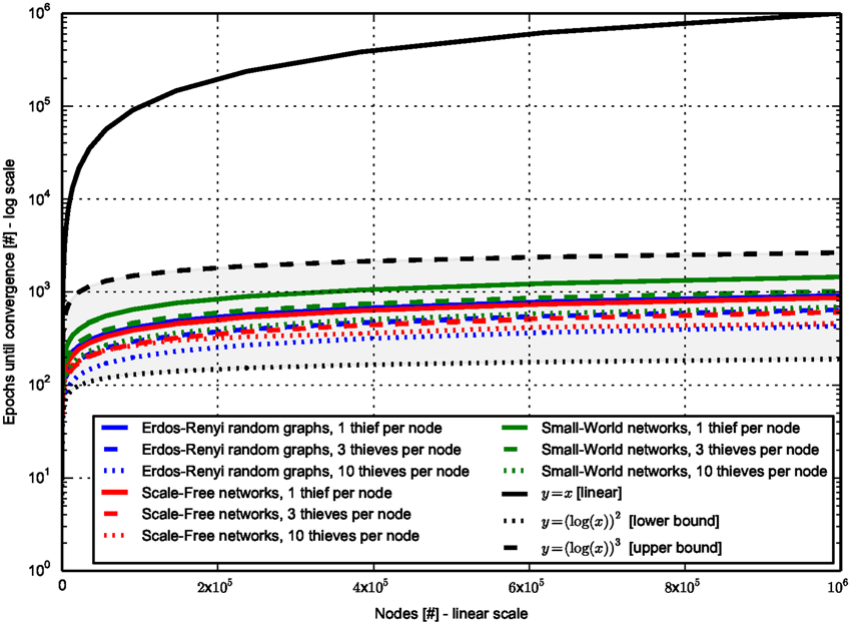
GOT scalability. The plot shows the number of epochs needed by GOT to converge. For each network used, the number of edges is between 5 and 10 times bigger than the number of nodes. Independently of the network model, or the number of agents used per node (i.e. 1, 3, or 10), GOT convergences in a number of epochs empirically lower-bounded by *log*^2^|*V*| and upper-bounded by *log*^3^|*V*|, which is on the polylogarithmic scale with respect to the total number of nodes in the network, |*V*|.

Empirically, we found that the number of epochs needed for convergence is on the polylogarithmic scale of the network size. Figure 3 depicts this sub-linearly relation for each network type. More exactly, the parallel time complexity of GOT convergence, *O*(*GOT*), is bounded by *log*^2^|*V*| < *O*(*GOT*) < *log*^3^|*V*|. Furthermore, due to the facts that each network node can run completely independently of the others, and the thieves can be emulated by messages transmitted between nodes, if we assume an environment where each node can do its own computations, then we can say that GOT is a fully distributed algorithm. Such environment can be offered, for instance, by all devices running a Facebook application. Even in a traditional parallel computing environment, a high level of parallelization can be achieved. For instance, the nodes could be split in disjunctive subsets, and each subset can run on a computing core. To our best knowledge, this represents a breakthrough compared to the state-of-the-art centrality algorithms which have at least a quadratic time complexity (see Table 1).

**Table 1:** Comparison of five centrality algorithms using different performance criteria (i.e. functional, computational efficiency, and accuracy). The bold values represent the best performer for specific performance criteria.

Algorithm	Functional Performance	Computational Efficiency Performance		Accuracy Performance
		Architecture	Time complexity	
GOT with *SC*_2_	**Nodes and Links**	**Fully Distributed**	***O*(*log***^**2**^|***V***|**)** < ***O*(*GOT*) < *O*(*log***^**3**^|***V***|**)**	83.4%
CFBC^31^	Nodes or Links	Centralized	*O*(*I*(|*V*| − 1) + |*V*||*E*|*log*|*V*|)	8.3%
BC^30^	Nodes or Links	Centralized	*O*(|*V*||*E*|)	8.3%
SOC^32^	Nodes	Partially Distributed	*O*(|*V*|^2^) < *O*(*SOC*) < *O*(|*V*|^3^)	0%
DACCER^21^	Nodes	**Fully Distributed**	n/a	0%

It has to be mentioned that computing the ranks of importance, Λ_*e*_, can be done by using a simple sorting algorithm on the ${\bar{{\rm{\Phi }}}}_{T}^{n}$ values. If GOT is run until the *SC*_2_ criteria is fulfilled than this sorting algorithm has to be executed in every epoch, according with Equation 3. We highlight that this overhead can be avoided by letting GOT to run for a fixed number of epochs, within the above discussed bounds. In this case the sorting algorithm can be executed just once, after GOT has been stopped, as demonstrated further in the *Performance on real-world networks* paragraph.

### GOT performance

We have assessed GOT both on simulated and real-world networks, against state-of-the-art centrality metrics, i.e. Betweenness Centrality (BC)^30^, Current Flow Betweenness Centrality^31^ (CFBC), DACCER^21^, and Second Order Centrality^32^ (SOC), as detailed in Methods.

**Figure 4:**
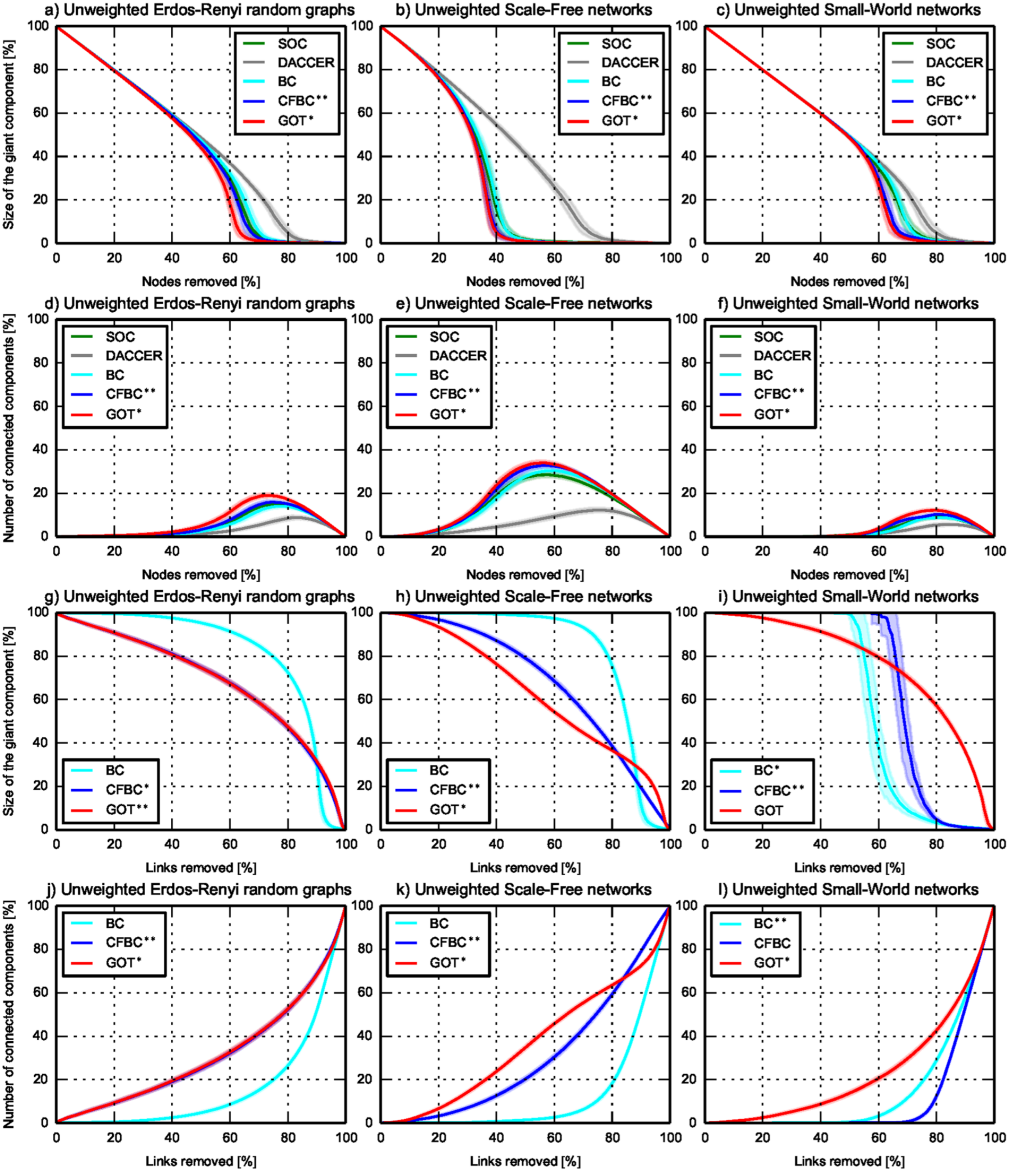
GOT accuracy - random generated unweighted networks. The evolution of the size of the giant component and of the number of connected components with mean (the straight line) and standard deviation (the shadow area) in unweighted networks during the NRP procedure, averaged over 100 networks in each subplot. The y-axes give figure of merit, while the x-axes represent percentage of node and links removals, respectively. In the top subplots, nodes centrality is assessed, while in the bottom subplots, the links centrality is evaluated.

**Figure 5:**
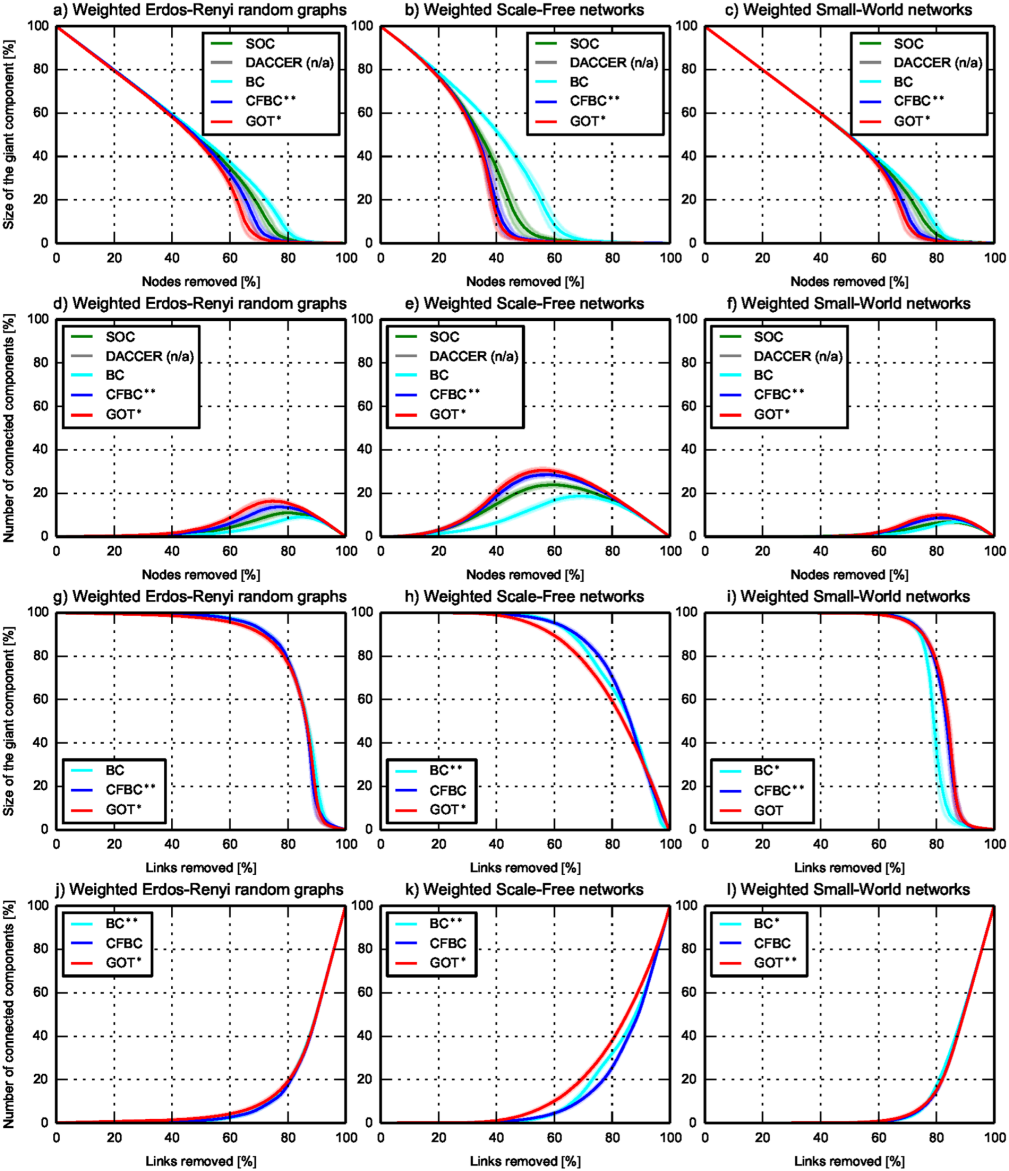
GOT accuracy - random generated weighted networks. The evolution of the size of the giant component and of the number of connected components with mean (the straight line) and standard deviation (the shadow area) in weighted networks during the NRP procedure, averaged over 100 networks in each subplot. The y-axes give figure of merit, while the x-axes represent percentage of node and links removals, respectively. In the top subplots, nodes centrality is assessed, while in the bottom subplots, the links centrality is evaluated.

To assess GOT’s accuracy in identifying the correct node centrality (while validating *SC*_2_), we used three classes of simulated networks: Erdös-Rényi Random Graphs, Scale-Free and Small-World networks. For each class, we randomly generated 100 weighted networks with weights generated randomly between 1 and 10, and 100 unweighted networks. Each network had 1,000 nodes and between 4,500 and 5,500 links. Comparing GOT to the literature was tricky, because nobody so far has managed to compute node and link centrality rankings simultaneously, as we do. We compared to two centralized methods, Brandes’ algorithm^30^ for Betweenness centrality and Current flow betweenness centrality^31^, which have variants for vertices and edges. We ran these multiple times to allow the comparison with GOT. Also, we compared GOT with two decentralized algorithms, DACCER^21^ and Second order centrality^32^, for nodes centrality. DACCER and SOC do not have variants for links centrality, and DACCER is not capable to assess nodes centrality in weighted networks. For GOT, we set 1 thief and ${{\rm{\Phi }}}_{0}^{n}\mathrm{=1000}$ vdiamonds per node and we ran the algorithm until *SC*_2_ convergence was achieved. To assess the accuracy of all metrics used, we used the NRP procedure^32^ (as detailed in Methods). Figure 4 and 5, and Table 2 depict the generality of GOT, which has a better accuracy than all the other centrality metrics for nodes, while for links it outperforms its competitors in 8 of 12 scenarios, staying very close to the best performer (BC or CFBC) in the remaining 4 scenarios. But we should note that BC and CFBC are only used to compare centrality accuracies - these are centralized algorithms and would not scale in massive-scale networks (which is the ultimate goal of GOT). In all scenarios, *SC*_2_ was fulfilled on average after 274 ± 45 epochs, this being within the previous discussed bounds. More than that, in both figures, it can be observed that GOT performs better because it is capable to discover well the centrality of the medium important nodes and links, while the other algorithms fail to do that.

**Table 2:** Experiments summary. Area under the curve (AUC), rounded to the nearest integer, computed for each metric from each subplot from Figs 4–6. The bold values represent the best performer for that specific scenario, while “n/a” means that the metric is not suitable for that specific scenario.

				SOC	DACCER	BC	CFBC	GOT
Random generated unweighted networks (Fig. 4)	Erdos	Nodes centrality	Giant size Components number	4293 486	4625 267	4348 453	4237 530	**4086** **668**
		Links centrality	Giant size Components number	n/a n/a	n/a n/a	8185 1574	**6843** 3129	6853 **3141**
	Scale-free	Nodes centrality	Giant size Components number	2960 1386	4167 571	2987 1433	2823 1569	**2794** **1620**
		Links centrality	Giant size Components number	n/a n/a	n/a n/a	8308 1277	6888 3042	**6362** **3633**
	Small- World	Nodes centrality	Giant size Components number	4447 272	4655 167	4472 252	4312 306	**4257** **365**
		Links centrality	Giant size Components number	n/a n/a	n/a n/a	**6038** 1484	7001 1127	7645 **2322**
Random generated weighted networks (Fig. 5)	Erdos	Nodes centrality	Giant size Components number	4501 354	n/a n/a	4659 258	4360 453	**4219** **566**
		Links centrality	Giant size Components number	n/a n/a	n/a n/a	8413 1262	8358 1257	**8287** **1340**
	Scale-free	Nodes centrality	Giant size Components number	3204 1199	n/a n/a	3803 841	2974 1392	**2917** **1479**
		Links centrality	Giant size Components number	n/a n/a	n/a n/a	8301 1547	8380 1444	**8073** **1837**
	Small-World	Nodes centrality	Giant size Components number	4636 191	n/a n/a	4732 162	4511 244	**4456** **280**
		Links centrality	Giant size Components number	n/a n/a	n/a n/a	**7907** **1208**	8231 1177	8263 1187
Real-World networks (Fig. 6)	Dolphins	Nodes centrality	Giant size Components number	3643 1030	3527 1228	2490 1875	2400 1971	**2272** **2344**
		Links centrality	Giant size Components number	n/a n/a	n/a n/a	5094 3691	**4172** 3946	5625 **4244**
	Internet	Nodes centrality	Giant size Components number	179 4217	1020 3034	180 4631	**163** 4577	179 **4641**
		Links centrality	Giant size Components number	n/a n/a	n/a n/a	5164 4289	4111 3685	**4027** **5972**
	High Energy	Nodes centrality	Giant size Components number	n/a n/a	n/a n/a	654 2789	n/a n/a	**649** **3291**
		Links centrality	Giant size Components number	n/a n/a	n/a n/a	3390 4645	n/a n/a	**3299** **6458**

### Performance on real-world networks

**Figure 6:**
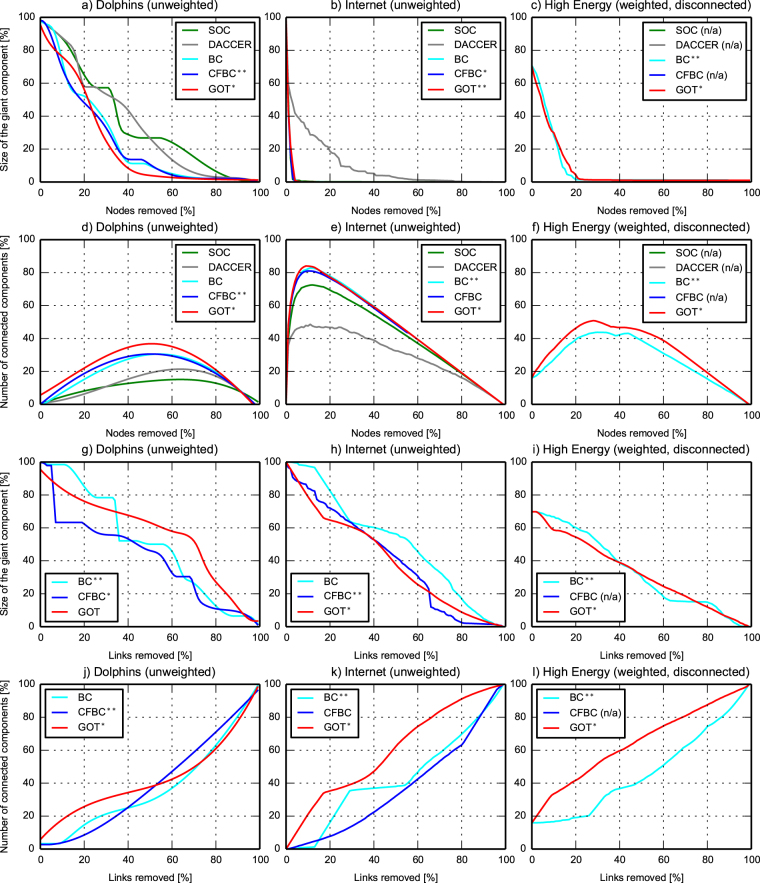
GOT accuracy - real-world networks. The evolution of the size of the giant component and of the number of connected components during the NRP procedure in three real-world networks: Dolphins (62 nodes, 159 links, unweighted), Internet (22963 nodes, 48436 links, unweighted), and High Energy (8361 nodes, 15751 links, weighted, disconnected). The y-axes give figure of merit, while the x-axes represent percentage of node and links removals, respectively. The top subplots depict the performance of nodes centrality metrics. The bottom subplots show the links centrality metrics.

We have validated GOT using three real-world networks (from different domains): the “Dolphins social network”, an undirected social network of the most frequent associations between a community of 62 dolphins living in Doubtful Sound, New Zealand^33^; the “Internet”, a symmetrized snapshot of the structure of the Internet created by Mark Newman from BGP tables posted by the University of Oregon in 2006; and the “High Energy” theory collaborations, a weighted disconnected network with the co-authorships between scientists posting preprints on the High-Energy Theory E-Print Archive between 1 January 1995 and 31 December 1999^34^. For GOT, we set 1 thief and ${{\rm{\Phi }}}_{0}^{n}=|V|$ vdiamonds per node and we ran it for *log*^2^|*V*| epochs (i.e. the lower bound of GOT with *SC*_2_) to avoid the overhead introduced by the *SC*_2_ computing. By using the same NRP procedure as before, Fig. 6 shows that GOT achieves a better accuracy than the other approaches in 10 out of 12 situations, while in the other 2 cases it stays very close to the best performer (CFBC) - again, CFBC is used only for comparison, being a centralized algorithm which would not be usable in massive-scale networks.

We emphasize that in the case of the “Internet” network, which was the biggest real-world network used in this paper (i.e. 22,963 nodes, 48,436 links) a Python sequential implementation of GOT ran in 88 seconds and assessed both, nodes and links centrality, at the same time, while the cumulative times for the next two performers, BC and CFBC using their NetworkX^35^ implementations, were 6,322 seconds and 66,977 seconds, respectively. These running times are at least two orders of magnitude larger than GOT. DACCER and SOC, using our own Python implementation, were a bit faster than BC and CFBC, and ran in 574 and 3,213 seconds, respectively, but their accuracy was much lower. Besides that, they were able to compute just nodes centrality.

The “High Energy” network was particularly interesting to show another singular feature of GOT: its ability to compute centrality in disconnected networks. This is not possible with existing distributed methods, so we use the centralized algorithm BC for the sake of performance comparison.

As a curiosity, looking at the “High Energy’’ network we found that prof. Jan Ambjorn was the most important researcher. Considering that this database was 17 years old, we found a strong correlation of GOT results with a recent Google scholar profile of prof. Jan Ambjorn (i.e. 16,194 citations, 68 h-index) on 22 nd May 2017. We can then speculate that centrality algorithms may even be used to make future extrapolations on networks.

## Discussion

GOT is a new approach to profiling complex networks using a fully decentralized method. It outperforms state-of-the art algorithms on three different performance criteria (i.e. functional, accuracy, and computational efficiency), as summarized in Table 1. Functionally, it is capable of assessing at the same time nodes and links importance in weighted, unweighted or disconnected networks. More than that, it outperforms state-of-the art algorithms in terms of accuracy, being capable to accurately capture the underlying relations between the network elements and to detect well all shades of centrality, including the most difficult entities - i.e. the one of medium importance. All of these are detailed in Table 2, which summarizes all the accuracy experiments by computing the area under the curve for each metric from each subplot of Figs 4–6. Overall, GOT was the best performer in terms of accuracy in 30 out of 36 scenarios, while in the remaining 6 it was the second best performer or very close to the best performers - but these are centralized, thus unscalable methods.

Besides that, in terms of computational complexity, GOT is much faster and scalable (in terms of both number of nodes and number of links) compared to existing methods. The worst-case implementation of GOT is sequential (i.e. it emulates all network actions in sequence in a single computer). Yet this is bounded up by *O*(|*V*|*log*^3^|*V*|), which is much faster than the next three followers in terms of accuracy BC, CFBC, and SOC. These have computational complexity of *O*(|*V*||*E*|)^30^, *O*(*I*|*V*| − 1) + |*V*||*E*|*log*|*V*| (where *O*(*I*|*V*| − 1) is the time necessary to compute the inverse Laplacian)^36^, and at least *O*(|*V*|^2^)^32^, respectively.

Another computational aspect which has to be considered is given by the randomness of GOT. Thus, as usual for stochastic algorithms, the best practice would be to run GOT many times on the same network and to take the statistical average into consideration. However, in practice, we found out that GOT is very stable and by running it just once on a particular network it offers very good results in terms of accuracy performance. This stability is best reflected by the very small standard deviations (the shadow areas) from Figs 4 and 5, where the results of each subplot are computed as an average over 100 random generated networks, on each of these networks GOT being run just once.

Even more strikingly, when GOT is implemented in distributed systems, its execution will proceed in parallel across all nodes. This natively decentralized version of GOT has a parallel time complexity on the polylogarithmic scale with respect to the number of nodes in a network. This makes it suitable to perform real-time analysis of very large-scale networks with billions of nodes, easily identifiable in the big data era, such as Facebook (in the range of 1.000.000.000 nodes) or the Internet of Things (expected to expand to an order of 1 trillion of nodes within the next few years).

To give an impression of the significance of the computational capability at hand, let us consider what GOT could achieve in a 1 trillion Internet of Things network of the near future. Assuming that each device would run GOT and would be able to transmit one message per millisecond. The scalability figures given above, would lead to a complete computation of all node and link ranks in a timespan comprised between 0.8 seconds (given by the lower bound of GOT with *SC*_2_) and up to 22 seconds (given by the upper bound of GOT with *SC*_2_). By comparison, if we were to use the state-of-the-art parallel processing algorithms of today on powerful computers, it would take at least several weeks of continuous computation to achieve comparable results. This places GOT in a much better position in terms of performing real-time centrality computations on massive-scale networks, being able to tackle not only scale but also network dynamics.

Concretely, GOT is more accurate and much more faster than the most used centrality metrics. Thus, we foresee that it will start replacing those metrics in a number of real-world problems where the correct and efficient identification of nodes and links centrality is essential: in biological neural networks^2–4^, in cosmic structures^5^, in biological networks^7^, for viruses spreading and containing^16^, to identify the people or the news capable to influence opinions the most in social networks^17,37^, to protect computer systems from cyber-attacks^18^, and so on.

In this paper we introduce a new viewpoint to understand and model complex networks, which overlays a homogeneous artificial system over a network to unveil its hidden properties. We propose a novel algorithm to compute centrality in networks, dubbed GOT. We show that GOT can compute all node and link centralities, treated together, in a polylogarithmic time with respect to the number of nodes in the network. GOT has the computational simplicity of nature-inspired swarm algorithms, while performing human-behaviour like computations^23^ (namely, egoistic behaviour). We demonstrate on thousands of simulated networks with different types of topologies, and on real-world networks, that GOT can compute the whole range of link and node strengths of any complex network, while being more accurate, much faster, scalable and technologically viable than the state-of-the-art centrality metrics. Moreover, we have also used it to confirm well-established findings about a non-obvious behaviour of natural networks^26^. Natively, GOT permits to investigate much larger networks, which are not tractable with current algorithms - for instance GOT would require less than 9 seconds to compute the centrality of the one-billion network formed by all Facebook user devices. The latter is one near future research direction that we would like to take. Another direction is to try to replace the sorting algorithm which computes the ranks of importance, Λ_*e*_, with a decentralized GOT extension which makes use of network statistics collected by thieves and vdiamonds with memory. Also, we intend to make a formal mathematical proof to show that GOT is as a stochastic process which has its stationary state.

To conclude, we anticipate that our approach may lead to advances in various research fields for which nodes and links centrality is of crucial importance^2–13^. Thus, we consider that our viewpoint will start a novel class of methods in network science which natively incorporate the primordial property of real-world networks, i.e. decentralization, and which may change our understanding about the natural and human-made complex systems modelled by networks.

## Methods

### Game of Thieves

#### Thieves behavior

In the paper, we have presented the intuitions and the main flow of the Game of Thieves (GOT) approach, and how it can be used to compute the centrality of vertices and edges in a graph *G* = (*V*, *E*), where (*V*) is the set of vertices, and (*E*) is the set of edges. A key ingredient in the success of GOT is the behavior of thieves (the agents) within the network. Before going into details, let us add the following notations: Γ^*n*^ is the set of nodes that are connected by a link with node *n*, ∀*n* ∈ *V*; with Ω^*nm*^ ≥ 0 the weight of the link which connects the nodes *n* ∈ *V* and *m* ∈ *V*; and with *Υ*_*a*_ a dynamic list with the nodes visited by thief *a*, useful to keep the path of *a* in his search for vdiamonds.

So, a thief *a* in the “empty” state will always perform successively the following operations in any epoch *e*:

- It randomly picks a node *m* ∈ Γ^*n*^, where *n* is its actual location, with the following probability ${p}_{a}^{nm}=\frac{{{\rm{\Omega }}}^{nm}}{{\sum }_{v\in {{\rm{\Gamma }}}^{n}}{{\rm{\Omega }}}^{nv}}$; and it moves to node m. It is clear that unweighted networks are just a particular case of weighted networks, by setting the weights of all links from the networks to 1.
- If *m* ∈ Υ_*a*_ then all the nodes situated after *m* in the list are removed from Υ_*a*_, to avoid the apparition of cycles in the list.
- If *m* ∉ Υ_*a*_ then *m* is added to the end of Υ_*a*_.
- If node *m* has vdiamonds then the thief *a* takes one and it changes his state to “loaded”, while node *m* decreases ${{\rm{\Phi }}}_{e}^{m}$ by one vdiamond.
- At the same time, a thief *a* in the “loaded” state will always perform successively the following operations in any epoch *e*:
- It moves from the last node *n* from Υ_*a*_, which is his actual location, to the last but one node *m* from *Υ*_*a*_, and after that it removes *n* from Υ_*a*_.
- Link *l* from *n* to *m* increases ${{\rm{\Psi }}}_{e}^{l}$ by one.
- If *m* is the home node of *a*, the thief unloads the vdiamond, and sets his state to “empty”, while node *m* increases ${{\rm{\Phi }}}_{e}^{m}$ by one vdiamond.

**GOT algorithm**. The algorithm is detailed below.

**Algorithm 1:**
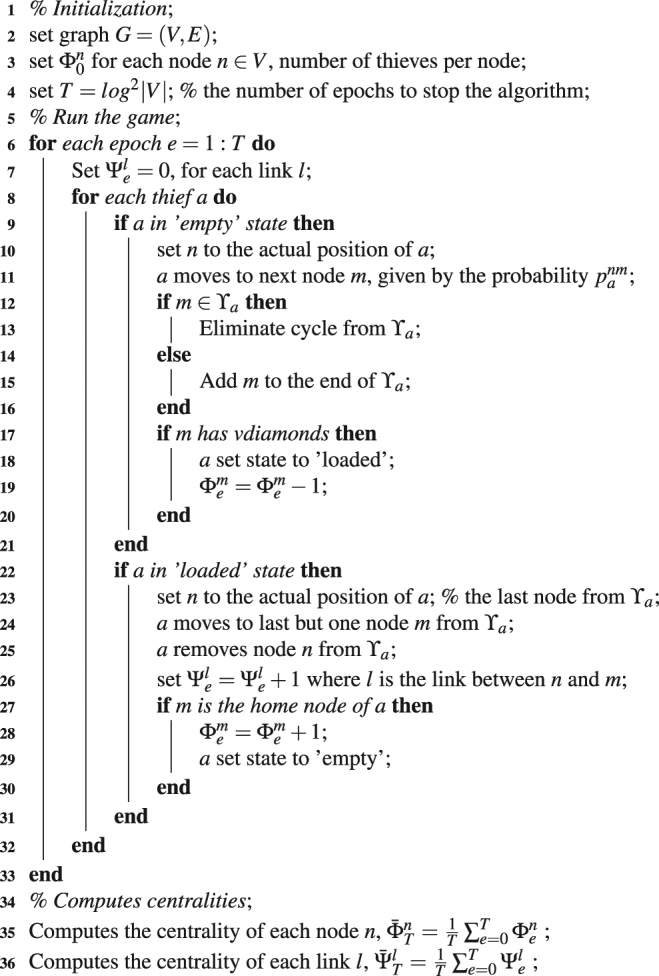
Game of Thieves (GOT) algorithm.

#### GOT optimal parameter choice

In total, GOT has three parameters: i.e. the number of epochs to run the game, the initial amount of vdiamonds which have to be set in each node, and the number of thieves in each node. In terms of accuracy, these parameters do not affect the algorithm performance, if the game is ran until the *SC*_2_ criteria is fulfilled. To clarify, *SC*_2_ represents an equilibrium state of GOT as a stochastic process. Once GOT arrives in this state, the above mentioned three parameters do not affect any-more the nodes and links ranks of importance. Throughout the experimental section of the paper, we evaluated the quality of these ranks given by GOT when the *SC*_2_ criteria was fulfilled.

Thus, we studied the three parameters just in term of computational efficiency and how they can affect (or delay) GOT to reach the *SC*_2_ criteria. Previously, we demonstrated that independently of the network size GOT converges to *SC*_2_ in a bounded number of epochs. So, we consider a safe practice to set the number of epochs to run the game to the lower bound of *SC*_2_, *O*(*log*^2^|*V*|), if one needs the results faster, or to the upper bound, *O*(*log*^3^|*V*|), if a better accuracy is needed. To find the best value for the initial amount of vdiamonds per node, we performed extra experiments on different network types and sizes. We found that this parameter does not significantly affect the convergence time of the algorithm if it is set to non trivial values, e.g. 1, 2, 3 vdiamonds per node. Our experiments showed us that best practice is to set this parameter to the total number of nodes in the network. We should, in fact, mention that the initial value of vdiamonds is not the crucial one, since it has negligible computational costs. Finally, we have analyzed how the number of thieves per node influences the number of epochs needed by the algorithm to converge considering different network types and sizes. In all cases, independently on the number of thieves, the game converged within the bounds of *SC*_2_. To conclude, we consider that by setting just one thief per node is enough, due to the fact that it achieves fast convergence time, independently of the cases studied, while being the fastest option in terms of the total number of messages exchanged in the network.

#### Centrality in complex networks

Centrality is a measure to assess how important individual nodes (or links) are in a network and how they can affect their neighborhood or even the whole network. However, there is no clear way to define “centrality” in graphs. In the literature, there are several methods to calculate node’s centrality, each one focused on specific features. Broadly, there are two main approaches: centralized and decentralized methods. We exemplify these approaches, through four state-of-the-art centrality metrics, as summarized in Table 1.

#### Betweenness Centrality (BC)

BC and its variants are among the most utilized metrics to assess the nodes’ importance^38^. It quantifies how a node lies on the path between other nodes. Formally, for a node *n*∈***V***, where *V* is the set of all nodes, this can be written as:

4 $${C}_{be}(n)=\sum _{w,u\in {\rm{V}}}\frac{{\sigma }_{w,u}(n)}{{\sigma }_{w,u}}$$

where *σ*_*w*,*u*_(*n*) represents the number of shortest paths from node *w* to node *u* which pass through the node *n*, and *σ*_*w*,*u*_ represents the total amount of shortest paths from *w* to *u*. The computational complexity of the original algorithm is ${\mathscr{O}}({n}^{3})$, making it unsuitable for large networks. For this reason, in the last period, several BC approximations have been proposed (see^30^ and references therein).

#### Current Flow Betweenness Centrality (CFBC)

It was proposed in^31^, and is inspired to how the electric current flows into an electric network. In comparison to BC, CFBC does not make the assumption that only the shortest paths are important to compute the node centralities. It considers all the possible paths in a network, by making use of random walks. In general, CFBC is considered to reflect centrality more accurately than BC, but it is slower.

#### Second Order Centrality (SOC)

It is a novel form of node’s centrality metric, calculated in a decentralized way, and proposed by Kermarrec *et al*. in^32^. The algorithm is based on a random walk in the graph, which starts from a random chosen node, and runs continuously. After the random walk has visited all nodes at least three times, the standard deviation of the number of steps required to reach each of the nodes is computed. The authors demonstrate why this value reflects the centrality of nodes.

#### DACCER

It is a decentralized algorithm to measure the centrality of nodes in networks, proposed by Wehmuth and Ziviani in^21^. The main idea is that each node is computing its own centrality, based on the information acquired from its vicinity. The authors showed that a two-hop vicinity reflects well the closeness centrality.

### Evaluation metric - Node Removal Procedure (NRP)

**Figure 7:**
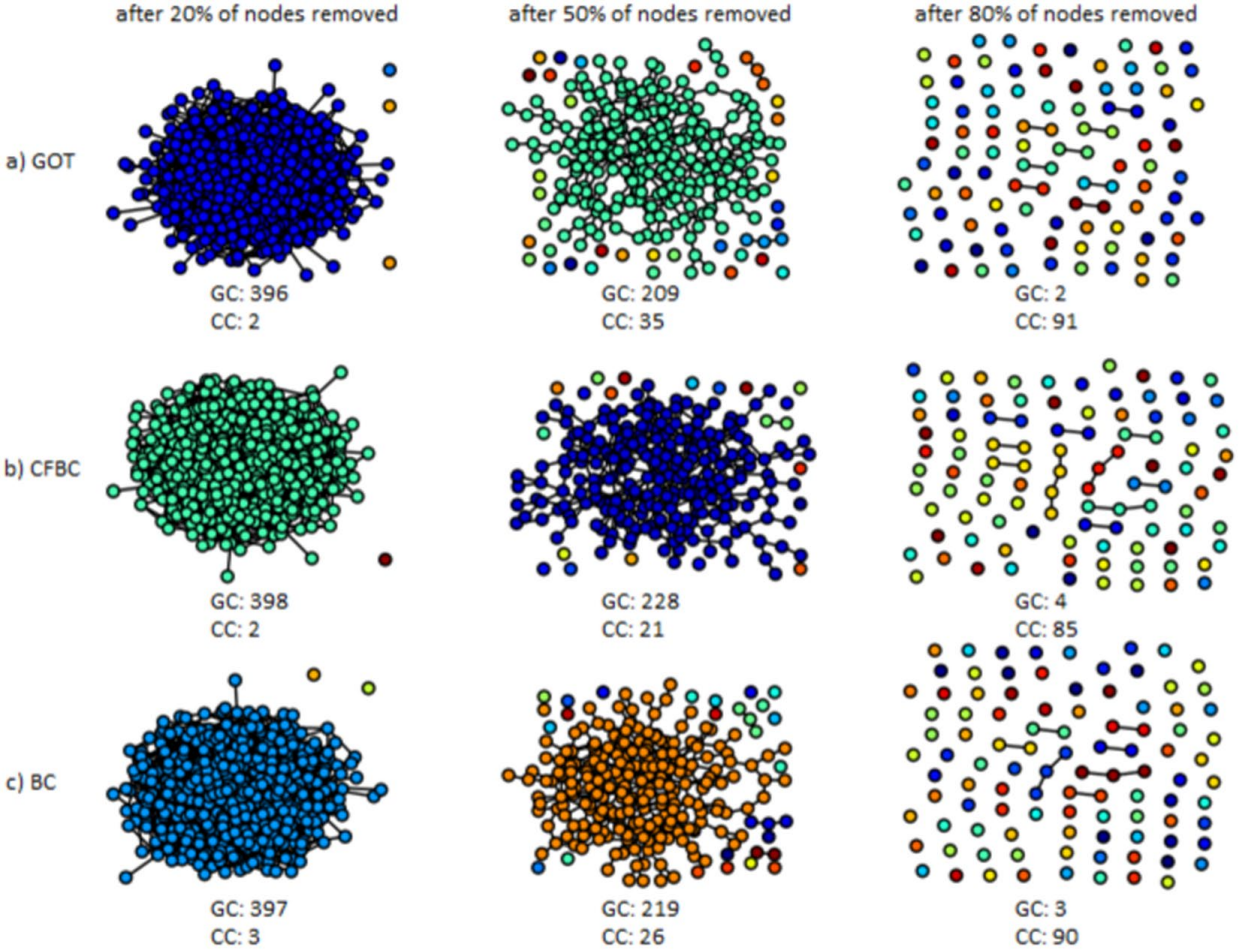
NRP procedure - nodes. Snapshots during the NRP procedure for nodes in a random generated network with 500 nodes. At the bottom of each subplot, the number of connected components (CC) and the size of the giant component (GC) are shown.

**Figure 8:**
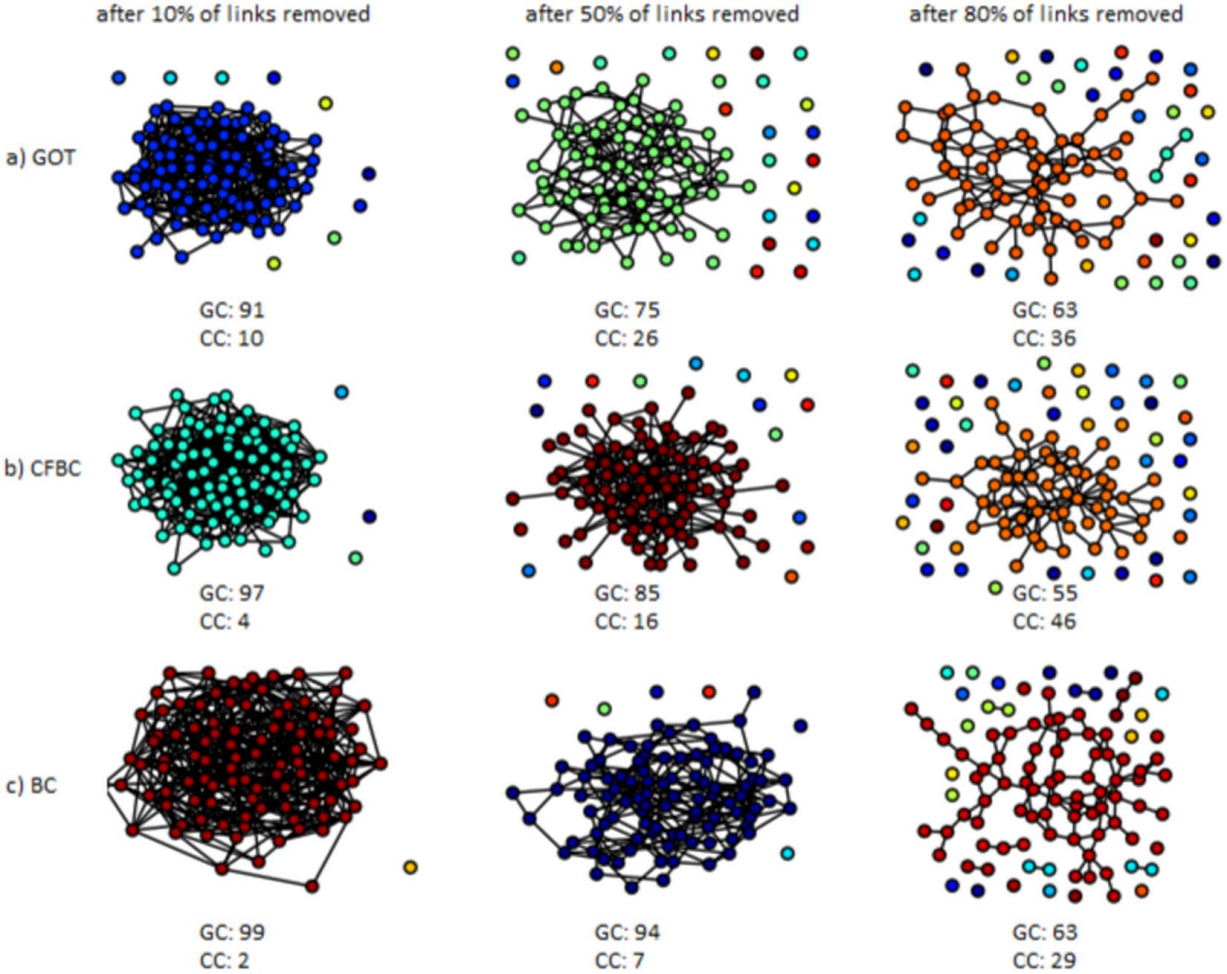
NRP procedure - links. Snapshots during the NRP procedure for links in a random generated network with 100 nodes. At the bottom of each subplot, the number of connected components (CC) and the size of the giant component (GC) are shown.

In the experiments, we have used a standard procedure to assess the accuracy of the nodes centrality metrics, namely the Node Removal Procedure (NRP)^32^, as described next. After a centrality metric assigns scores for each node of the graph, all the nodes are sorted according to their scores, starting with the most important one, and ending with the less important one. Furthermore, the nodes from this sorted list are removed one by one from the graph, and after each removal the size of the Giant Component (GC) and the number of Connected Components (CC) in the remaining graph are measured. A node centrality metric is considered to be better if the number of connected components is as big as possible, while the size of the giant component is as small as possible, during this NRP procedure. Similarly, NRP can be applied for links, if the links are sorted according with their importance and after that they are removed one by one. In Fig. 7, we have illustrated some snapshots during the NRP procedure for nodes in a random network with 500 vertices. In Fig. 8 we have illustrated the NRP procedure for links in a random network with 100 vertices.

### Implementation

For all the experiments performed in this paper we used Python and the NetworkX library^35^. Furthermore, for BC and CFBC we used the standard implementations offered by the aforementioned library, while GOT, DACCER and SOC were fully implemented by us. Moreover, we used NetworkX to generate the simulated networks, to work with the real-world networks under scrutiny, and to compute the size of the giant component and the number of connected components during the NRP procedure. The hardware platform utilized was a typical desktop computer (i.e. Intel Core i7, 32 GB RAM).

### Data Availability

The source code will be available online after the acceptance of the paper, while the data will be available from the authors upon request.

## References

[1] Strogatz SH (2001). Exploring complex networks. Nature.

[2] Pessoa L (2014). Understanding brain networks and brain organization. Physics of Life Reviews.

[3] Stam CJ (2014). Modern network science of neurological disorders. Nature Reviews Neuroscience.

[4] Fornito A, Zalesky A, Breakspear M (2015). The connectomics of brain disorders. Nature Reviews Neuroscience.

[5] Hong S, Dey A (2015). Network analysis of cosmic structures: Network centrality and topological environment. Monthly Notices of the Royal Astronomical Society.

[6] Wuchty, S. & Uetz, P. Protein-protein interaction networks of e. coli and s. cerevisiae are similar. *Scientific Reports* **4** (2014). 10.1038/srep07187. 10.1038/srep07187 PMC5384207 25431098

[7] Jeong H, Mason SP, Barabási A-L, Oltvai ZN (2001). Lethality and centrality in protein networks. Nature.

[8] Kitsak M (2010). Identification of influential spreaders in complex networks. Nature Physics.

[9] Freeman LC (1978). Centrality in social networks conceptual clarification. Social Networks.

[10] Iranzo, J., Buldú, J. M. & Aguirre, J. Competition among networks highlights the power of the weak. *Nature Communications* **7**, 10.1038/ncomms13273 (2016). 10.1038/ncomms13273 PMC5114531 27841258

[11] Crucitti P, Latora V, Porta S (2006). Centrality measures in spatial networks of urban streets. Phys. Rev. E.

[12] Mocanu, D. C., Exarchakos, G. & Liotta, A. Node centrality awareness via swarming effects. In *2014 IEEE International Conference on Systems, Man, and Cybernetics (SMC)*, 19–24 (2014).

[13] Albert R, Jeong H, Barabási A-L (2000). Error and attack tolerance of complex networks. Nature.

[14] Mocanu DC, Mocanu E, Nguyen PH, Gibescu M, Liotta A (2016). A topological insight into restricted boltzmann machines. Machine Learning.

[15] Brockmann D, Helbing D (2013). The hidden geometry of complex, network-driven contagion phenomena. Science.

[16] Pei S, Makse HA (2013). Spreading dynamics in complex networks. Journal of Statistical Mechanics: Theory and Experiment.

[17] Aral S, Walker D (2012). Identifying influential and susceptible members of social networks. Science.

[18] Wang P, González MC, Hidalgo CA, Barabási A-L (2009). Understanding the spreading patterns of mobile phone viruses. Science.

[19] Lawyer, G. Understanding the influence of all nodes in a network. *Scientific Reports* **5**, 10.1038/srep08665 (2015). 10.1038/srep08665 PMC4345333 25727453

[20] Borgatti SP (2005). Centrality and network flow. Social Networks.

[21] Wehmuth K, Ziviani A (2013). Daccer: Distributed assessment of the closeness centrality ranking in complex networks. Computer Networks.

[22] Bonabeau E, Dorigo M, Theraulaz G (2000). Inspiration for optimization from social insect behaviour. Nature.

[23] Sanfey AG (2007). Social decision-making: Insights from game theory and neuroscience. Science.

[24] Exarchakos G, Antonopoulos N (2013). Cooperative stalking of transient nomadic resources on overlay networks. Future Generation Computer Systems.

[25] Witten TA, Sander LM (1981). Diffusion-limited aggregation, a kinetic critical phenomenon. Phys. Rev. Lett..

[26] Saavedra S, Stouffer DB, Uzzi B, Bascompte J (2011). Strong contributors to network persistence are the most vulnerable to extinction. Nature.

[27] Erdös P, Rényi A (1959). On random graphs i. Publicationes Mathematicae (Debrecen).

[28] Barabási A-L, Albert R (1999). Emergence of scaling in random networks. Science.

[29] Watts DJ, Strogatz SH (1998). Collective dynamics of ‘small-world’ networks. Nature.

[30] Brandes U (2001). A faster algorithm for betweenness centrality. Journal of Mathematical Sociology.

[31] Newman MJ (2005). A measure of betweenness centrality based on random walks. Social Networks.

[32] Kermarrec, A.-M., Le Merrer, E., Sericola, B. & Trádan, G. Second order centrality: Distributed assessment of nodes criticity in complex networks. *Computer Communications* **34**, 619–628, Special Issue: Complex Networks (2011).

[33] Lusseau D (2003). The bottlenose dolphin community of doubtful sound features a large proportion of long-lasting associations. Behavioral Ecology and Sociobiology.

[34] Newman MEJ (2001). The structure of scientific collaboration networks. Proceedings of the National Academy of Sciences of the United States of America.

[35] Hagberg, A. A., Schult, D. A. & Swart, P. J. Exploring network structure, dynamics, and function using networkx. In Varoquaux, G., Vaught, T. & Millman, J. (eds) *Proceedings of the 7th Python in Science Conference*, 11–15 (Pasadena, CA USA, 2008).

[36] Brandes, U. & Fleischer, D. Centrality measures based on current flow. In *Proceedings of the 22Nd Annual Conference on Theoretical Aspects of Computer Science*, STACS'05, 533–544 (Springer-Verlag, 2005).

[37] Hu Y, Ji S, Feng L, Havlin S, Jin Y (2015). Optimizing locally the spread of influence in large scale online social networks. arXiv preprint arXiv.

[38] Brandes U (2008). On variants of shortest-path betweenness centrality and their generic computation. Social Networks.

