# Author Correction: Decentralized dynamic understanding of hidden relations in complex networks

**DOI:** 10.1038/s41598-018-24851-9

**Published:** 2018-04-30

**Authors:** Decebal Constantin Mocanu, Georgios Exarchakos, Antonio Liotta

**Affiliations:** 1 Department of Mathematics and Computer Science, Eindhoven University of Technology, Eindhoven, 5612 AP The Netherlands; 2 Department of Electrical Engineering, Eindhoven University of Technology, Eindhoven, 5612 AP The Netherlands; 3 Present Address: Department of Electronics, Computing and Mathematics, University of Derby, Derby, DE22 1GB UK

Correction to: *Scientific Reports* 10.1038/s41598-018-19356-4, published online 25 January 2018

This Article contains errors in the Methods section under the subheading ‘Data Availability’.

“The source code will be available online after the acceptance of the paper, while the data will be available from the authors upon request.”

should read:

“A proof of concept implementation of GOT can be found on https://github.com/dcmocanu/centrality-metrics-complex-networks, while the data is available from the authors upon request.”

